# Reducing Heavy Drinking Through the “Sober Curious” Movement in Australia: Protocol for a Mixed Methods Study

**DOI:** 10.2196/72631

**Published:** 2025-06-10

**Authors:** Paul Russell Ward, Michael Savic, Sarah MacLean, Belinda Lunnay, Antonia Lyons, Tonda Hughes, Kerry London, Gabriel Caluzzi, Simone Pettigrew, Amy Pennay, Samantha Meyer, Tristan Duncan, Nicole Lee

**Affiliations:** 1 Research Centre for Public Health, Equity and Human Flourishing Torrens University Australia Adelaide Australia; 2 School of Health and Social Development Monash University Melbourne Australia; 3 Centre for Alcohol Policy Research La Trobe University Melbourne Australia; 4 Centre for Addiction Research University of Auckland Auckland New Zealand; 5 Center for Sexual and Gender Minority Health Research Columbia University New York, NY United States; 6 Research and Innovation Office Torrens University Australia Adelaide Australia; 7 Centre for Food Policy The George Institute for Global Health Sydney Australia; 8 School of Public Health Sciences University of Waterloo Waterloo Canada; 9 Chief Executive Office Hello Sunday Morning Sydney Australia

**Keywords:** alcohol reduction, sober curiosity, public health, co-design, abstinence, alcohol, qualitative research

## Abstract

**Background:**

Alcohol consumption is a major public health problem. Its socially engrained nature adds complexity to designing successful reduction approaches. Rather than implementing another intervention, we will undertake a natural experiment on the “sober curious” movement, which gained momentum through social media influencers promoting the idea of reducing alcohol consumption for wellness. We focus on ways to reduce alcohol consumption, through sober curiosity, with 4 heavy-drinking population groups: male construction workers; lesbian, gay, or bisexual women; hospitality workers; and tertiary education students.

**Objective:**

Aim 1 analyzes the sober curious movement from the “supply side” using qualitative interviews with sober curious stakeholders and a citizen science study of social media content with the 4 case study groups. We will also undertake citizen science and social media studies with a representative sample of the population. Aim 2 examines the sober curious movement from the “demand side” using qualitative interviews with the 4 case study groups to investigate their knowledge and attitudes toward sober curiosity. We will also undertake a representative national survey and ethnography with a representative sample of the population. For aim 3, we will develop evidence-based interventions leveraging sober curiosity and using citizens’ juries, industry symposia, and policy symposia to develop feasible public health measures and options tailored to the needs of the 4 case study groups.

**Methods:**

The project involves 3 stages. Stage 1 will examine the supply side of alcohol-free products. A social media analysis of marketing by alcohol-free producers and distributors will generate an understanding of their techniques and population groups they target. In-depth interviews with producers will create evidence on the intentions behind making alcohol-free products available, their target market, and if and how they balance providing nonalcoholic products alongside alcohol. Stage 2 will be a qualitative study with 4 case study groups with high alcohol consumption: male construction workers; lesbian, gay, or bisexual women; hospitality workers; and tertiary education students. This stage will provide a deep understanding of the reasons for alcohol consumption, potential for alcohol-free product use, and possible interventions to sustainably reduce consumption. Stage 3 will involve deliberative symposia with non-alcoholic beverage producers and distributors, representatives from our case study groups, public health professionals, and policy makers to develop co-designed interventions for alcohol reduction.

**Results:**

This 3-year research protocol was funded by the Australian National Health and Medical Research Council via their Ideas Grants funding scheme (grant ID GNT2038211). The study will commence in July 2025. Human Research Ethics Committee approval has been granted.

**Conclusions:**

Our study will provide a template for interventions designed to enable reduced drinking within heavy-drinking social worlds with huge potential for scalability of knowledge, expanding the economic, environmental, social, and cultural benefits within and across Australia and internationally.

**International Registered Report Identifier (IRRID):**

PRR1-10.2196/72631

## Introduction

### The Research Problem and Significance

Alcohol consumption is a major public health problem, contributing to a myriad of health problems such as increased avoidable mortality, chronic illness, and accidents, in addition to social and economic problems (eg, lost days of work, domestic violence) at a cost to the Australian economy of AUD $66.8 billion a year [1]. Australia’s National Alcohol Strategy 2019-2028 aims for a 10% reduction in population-level alcohol consumption [2]. This reduction aim is difficult because consuming alcohol is socially acceptable, and heavy-drinking groups sustain population-level harms [3,4]. Indeed, the heaviest drinking 10% of the Australian population accounted for 54% of all alcohol consumed in 2019 [5]. The term “heavy-drinking” refers to levels exceeding the national alcohol guidelines [6] for long-term (>10 standard drinks per week) or single-occasion (>5 standard drinks on one occasion per month) risky drinking. This puts these particular heavy-drinking groups at higher risk of lifetime harm than other people. Urgent action is required to identify socially acceptable alcohol reduction options for heavy-drinking Australians [6].

This project will investigate the untapped potential for public health to leverage the momentum of the “sober curious” (SC) movement toward increasing the social acceptability of nondrinking. Prominent on social media, SC is a social movement that challenges the pervasiveness of alcohol in social contexts, emphasizes the health benefits of reduced alcohol consumption (or abstinence), and provides a series of supports and strategies to help realize these benefits. Our purpose is to ascertain if, how, and why (or why not) the SC movement influences heavy drinkers’ openness and ability to reduce alcohol consumption. It explores the leverage potential through two key aspects of the movement: SC programs and communities (eg, Hello Sunday Morning—associate investigator [AI] NL is Chief Executive Officer of this SC organization) and no or low-alcohol (termed NoLos) beverages, producers, and venues. Building on these insights, we will co-design and co-develop new alcohol reduction interventions that have relevance, consonance, and specificity among 4 heavy drinking “case study” groups: (1) male construction workers; (2) hospitality sector workers; (3) lesbian, gay, bisexual, transgender, queer, plus (LGBTQ+) women; and (4) tertiary education students (university and technical and further education [TAFE]) living in regional areas. To the best of our knowledge, this project is a world-first investigation of the diffusion (supply) and uptake (demand) of SC, aimed at reducing drinking among heavy-drinking groups by supporting long-term, sustainable, lower-risk drinking practices.

Although there has been reduced alcohol consumption at a population level, heavy drinking persists in our 4 case study groups:

- Male construction workers: Heavy drinking and “alcohol abuse” has been reported in the male construction industry [7], related to workplace culture and “masculine” group norms [8], where heavy drinking practices are normalized [7]. More than 60% of male construction workers regularly (monthly or more) consume ≥5 drinks in a single session, compared with 40% of men in general [9].
- LGBTQ+ women: Heavy drinking has been found across numerous countries among LGBTQ+ women [10-12] who often participate in alcohol-centric social contexts in order to establish friendships and gain social support [13,14]. They are more likely to drink heavily in these contexts [15]. There is a major gap in knowledge about developing alcohol interventions with and for LGBTQ+ women [11,16].
- Hospitality workers: Heavy drinking is prominent among the hospitality industry. Hospitality workers are 3.5 times more likely than other workers to consume alcohol and are more likely to attend work under the influence of alcohol than other workers [17]. In a recent study of hospitality workers in Victoria, around 75% regularly (monthly or more) consumed alcohol at risky single-session levels [18].
- Tertiary education students in regional areas: Young adults living in regional areas drink (on average) at heavier levels than those in urban areas [19,20]. For example, 62% of young adults in regional areas consumed >5 drinks on a single occasion per month compared with 47% in metropolitan areas [21]. Students are over-represented, indicated by 58% of young people with a certificate or diploma drinking at a risky level and those who finished year 12 drinking more than those who did not [6].

Current alcohol reduction interventions are not working for the 4 case study groups. Our approach differs from existing alcohol reduction approaches focused on individual consumption instead of the social context. It is essential to explore the possibility to use the current groundswell for SC to support urgent alcohol reductions by tailoring SC programs, communities, and NoLo beverages with and to Australia’s heaviest drinkers. The proposed project is a significant shift away from public health approaches focusing on deficits, which research evidence shows to be limited for reducing alcohol yet persist as a predominant philosophy underpinning current approaches [4,22].

This project represents a novel and time-critical opportunity. It responds to urgent calls to “change our heavy drinking culture” [2], provides an evidence base to support policy changes that increase the accessibility of NoLos, and informs the development of SC programs to meet the needs of heavy-drinking populations. Our research will achieve these outcomes by investigating modifiable group-level alcohol consumption practices, social norms, and identities rather than focusing on individual consumption patterns [16,21]. Our idea represents new territory for public health by focusing on the value and realization of positive nondrinking ways of living.

### Key Literature on Alcohol Consumption in Australia

Alcohol consumption in Australia is socially accepted and entwined in what many Australians think makes for pleasurable social engagements [23]. Current data show that 77% of the adult population drank alcohol in 2019 [24] and over 25% of adults exceeded the Australian alcohol guideline thresholds for single-occasion risky drinking in 2020-2021 [25]. Several population groups are at higher risk of harm due to heavy drinking and warrant urgent intervention [26]: male construction workers [7], LGBTQ+ women [11,16], hospitality sector workers [18], and tertiary education students in regional areas [19,27]. Given the persistence and harms of heavy drinking among these groups, urgent action is required to identify socially acceptable alcohol reduction options to reduce population-level harms. The SC approach is one option that is increasingly popular and is a real-world possibility. This project will co-develop and co-design options to use and extend aspects of the movement—programs and communities and NoLos—to support alcohol reduction within heavy-drinking groups. These comprise people who consume alcohol at levels exceeding the national alcohol guidelines for long-term (>10 standard drinks per week) or single-occasion risky drinking (>5 standard drinks on one occasion per month [6]), herein termed “heavy drinking.”

There are key shortcomings to current public health responses. Current public health responses to reducing alcohol consumption focus on individual risk messaging and behavior change, stripped from contexts, and the heavy drinking by particular groups persists. These shortcomings necessitate new targeted approaches that address the norms, identities, and practices that operate to sustain drinking.

Our previous work provides in-depth knowledge about the reasons other heavy-drinking groups (midlife women and urban young adults) consume alcohol [28-34] and provides the potential to leverage the SC movement to support alcohol reductions [35]. SC is a social movement encouraging wellness through alcohol reduction, focusing on normalizing nondrinking (or lighter drinking) and increased health consciousness. The SC movement also provides strategies or practices for effectuating and maintaining these transformations (eg, consumption of NoLos, alcohol-free spaces). The movement is prominent on social media, where social media influencers promote drinking in moderation and present it as pleasurable and beneficial [36]. SC represents an important shift away from Australia’s “culture of intoxication” [3], which is appropriated by the alcohol industry that profits most from heavy drinking. It emphasizes the health benefits of reducing alcohol consumption and prompts questions about the centrality of alcohol in social life. It fits well with other data suggesting increases in abstinence among young people are part of a broader normalization of nondrinking [37]. Although SC has had success in reaching younger, female, and middle-class groups [24], its potential for reaching and engaging other groups will be explored in the proposed study.

The potential for alcohol reduction in heavy-drinking groups is real. Awareness of the links between alcohol and health harms is growing, and increasing numbers of Australians are open to reducing drinking [38,39], with “health” given as the most common reason for reducing drinking [26,40]. This shift in heavy-drinking social norms demonstrates the potential for leveraging the momentum of the SC movement, whereby moderate or nondrinking is increasingly a plausible and health positive decision—for heavy drinkers specifically. Our proof of concept is our pilot studies [35], which found that the SC movement increased heavy-drinking, midlife women’s motivations and attitudes toward reducing alcohol [24].

There has been increasing availability of NoLos. The Australian Institute of Health and Welfare reported that, between 2016 and 2019, the proportion of ex-drinkers increased from 7.6% to 8.9% [41]. Sales of NoLos increased by 83% in the 12 months following Australia’s COVID-19 lockdowns in 2020 [35] and were predicted to increase by a further 24% by 2024 [42]. In April 2023, the South Australian Government announced an investment of Aus $2 million in a “No and Low Alcohol Trial Scale Research Facility, as part of its bigger NOLO Alcohol Program” [43]. This is a world-first, trial-scale NoLo facility. This investment recognizes the rapidly growing global NoLo wine market, worth an estimated AUD $1.6 billion in 2020 [43]. This shift in the market represents a huge potential to concurrently change the social contexts that encourage heavy drinking.

### Research Aims

#### Aim 1

Our first aim is to analyze the SC movement from the “supply side” using qualitative interviews with SC stakeholders and a citizen science study of social media content on NoLos and SC with the 4 case study groups to identify the reach and influence of social media and ways to support SC. PhD project 1 will additionally undertake citizen science and social media studies in a representative sample of the population.

#### Aim 2

Our second aim is to examine the SC movement from the “demand side” using qualitative interviews with the 4 case study groups to investigate their knowledge and attitudes toward SC. PhD project 2 will additionally undertake a representative national survey and ethnography in a representative sample of the population.

#### Aim 3

To address aim 3, we will develop evidence-based interventions for leveraging SC using citizens’ juries (the 4 case study groups), industry symposia (stakeholders linked to each case study group), and policy symposia to develop feasible public health measures and options tailored to the needs of the 4 case study groups.

### Conceptual Framework—Social Practice Theory and “Social Worlds”

The underpinning conceptual framework of the project is social practice theory (SPT) [44,45], which has been used extensively by chief investigators (CIs) PRW and BL [46,47], SM [31], and AL [34] and identified as critically important for examining how alcohol consumption behaviors develop, persist, and can be changed [48-50]. In their previous Australian Research Council (ARC)–funded study, CIs PRW, SM, and AL and AI AP applied SPT to interpret heavy-drinking, midlife women’s engagement with SC [35]. CI PRW has another ARC-funded study focusing on SPT around oral health in children [51,52]. Both have methodological and theoretical relevance to our proposed study.

SPT focuses on the shared *social practices*, including materials, meanings, and competencies, that frame and support alcohol consumption [53]. SPT provides a useful approach for examining alcohol practices in heavy-drinking social worlds. It stipulates that change can occur by addressing one or more of the elements that constitute drinking practices or practices that relate to heavy-drinking behavior (eg, work, eating, socializing). For instance, CI MS has shown how the social practices of “after work drinks” can be altered by shifting what it means to socially connect with colleagues and offering different ways of connecting that do not revolve around alcohol [18]. The 4 case study groups are exemplars of what CIs SM and MS, with AI AP, have conceptualized as heavy drinking “social worlds” [54], where drinking alcohol is an important aspect of shared norms, symbolisms, and activities, placing members of the groups at high risk of alcohol-related harm.

Considering how we might change the social practices shaping heavy-drinking social worlds allows greater scale of effect than does a focus on individuals alone, which has proven limited for reducing consumption behavior among heavy drinkers [4]. For example, in the social worlds of men in the *construction industry*, alcohol consumption is a central element of social inclusion, creating a sense of unity (“being one of the boys”) [55] and as an “act of friendship” [56], enabling men to support each other. In the social worlds of *LGBTQ+ women*, alcohol plays a key role in identity formation [57] and building relationships, and there are social expectations to drink in LGBTQ+ spaces [58]. Among *hospitality workers*, alcohol contributes to professional identities, cultivating fun atmospheres for patrons, social connection with workmates, and coping with the demands of busy shifts [18]. An SPT study of *tertiary students* showed how university activities and the symbolic aspects of drinking alcohol (eg, a rite of passage) bundle together to reinforce heavy consumption [14]. Overall, SPT provides a powerful framework for understanding how SC can be built into the social worlds of heavy-drinking groups.

### Pilot Studies That Informed the Proposed Study

Pilot study 1 included 90 interviews with midlife women (CI PRW’s ARC-funded study 2019-2023) to explore their logic and reasons for drinking alcohol and their preparedness for alcohol reduction. We found an openness to SC because it is increasingly popular in the broader community and to moderate drinking by consuming NoLos before or alongside alcohol in social situations [29,30,59,60]. This study focused specifically on midlife women, and the new study will build on it, extending to other groups of heavy drinkers and intersectional forms of oppression.

Pilot study 2 (led by CI BL) investigated factors that support women to be SC (ie, features of programs, forums, and networks) and reduce drinking [35], finding that middle-class women perceived the SC movement as optimistic and a form of empowerment and this enabled alcohol reductions. This study was only undertaken with midlife women, and this new study will build on those findings with new groups of heavy drinkers.

Pilot study 3 is a multicountry, cross-sectional survey (led by CI TH) with LGBTQ+ women in Australia (CIs PRW, SM, and BL), New Zealand (CI AL), Scotland, and the United States (CI TH) to understand barriers and facilitators to SC in this marginalized population group [61]. This pilot study includes cross-sectional surveys that will begin to describe the potential for SC programs to reduce alcohol consumption, but this new study will use novel qualitative methods to gain additional depth of understanding.

## Methods

### Ethical Considerations

Ethical procedures and approvals for all aspects of our study were sought and provided by Torrens University Human Research Ethics Committee (ID 0467). As with all mixed methods, ethical considerations include informed consent, anonymity, and confidentiality. The topic of alcohol consumption could also be sensitive. Our previous qualitative interview studies with diverse women in midlife (aged 45-64 years) has attuned us to issues pertaining to informed consent including detailing the potential risks and benefits of the study. Our approach in related studies has been to demonstrate empathic neutrality [62] in our style of engagement.

Each participant will have agency to decide the terms of their participation and the information they contribute. Other ethical practices include checking the suitability of the approach with participants before commencing data collection; allowing participants to nominate their preferred pseudonym, modes of contact, and preferred medium for data collection (phone, in-person, or telecommunication software); outlining assurances of confidentiality; and issuing honoraria to recompense their time and resources. Given the success of our previous studies at generating rich understanding and engaging women across long time periods or repeated interviews [47,63-65], we will use similar methods in this study.

### Research Design

**Figure 1:**
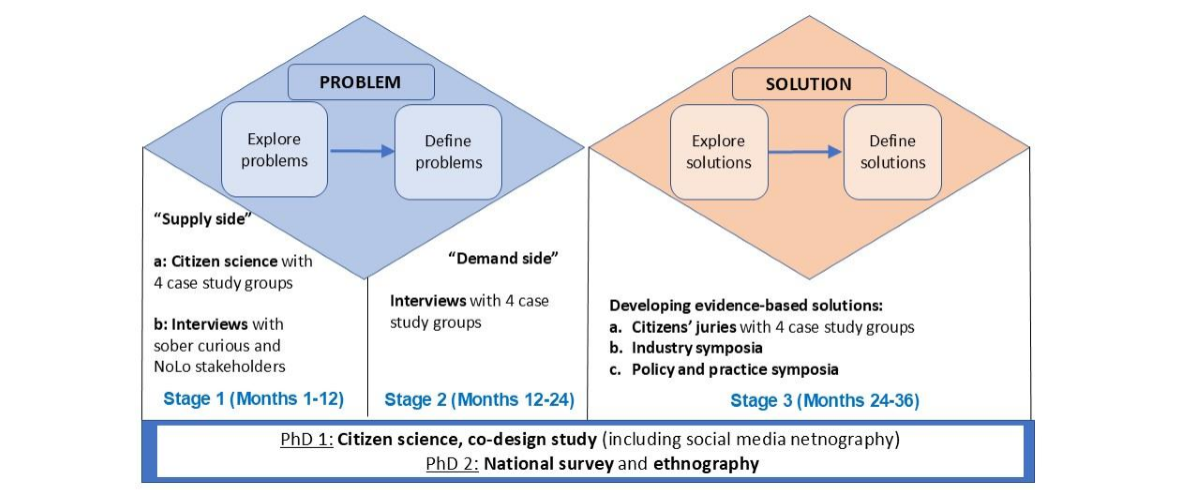
Research design and timeline. NoLo: no or low-alcohol.

The project will occur in 3 interrelated stages (see Figure 1) and follows an experience-based, co-design method [66,67] to investigate the “problems” with consumers (4 case study groups) and stakeholders then use their responses as ideas to devise and test public health “solutions” (programs and policy) that have relevance, consonance, and specificity to them—the intended end user. We will recruit participants for the project from all states and territories across Australia. We will also facilitate 2 PhD projects (funded by Torrens University) to add breadth to the study by examining the potential for SC within a broader cross-section of the Australian population.

The majority of data across all stages of the study will be qualitative, albeit from interviews, social media analysis, ethnographies, and different methods of consensus development. With participants’ consent, interviews and consensus groups will be audio-recorded and transcribed verbatim then deidentified and checked for accuracy. Although data analysis will differ slightly between studies, all data will be managed using NVivo v13 [68]. Analysis will follow the 3-step progressive method of precoding and conceptual and theoretical categorization developed by CI PRW and AI SM [69]. For interpretive validity, multiple researchers will independently pre-code samples of transcripts and field notes, social, or media and meet to compare coding and revise the coding frame.

### Stage 1a (Months 1 to 9, Led by CIs PRW, MSc, and AL and Linked to Aim 1)

This world-first study will analyze the context and intentions of the SC movement by including the supply side, with the view of leveraging the momentum of the movement to suit the social worlds of our 4 case study groups: male construction and hospitality workers, LGBTQ+ women, and tertiary education students living in regional areas. We will undertake a social media analysis (similar to analysis undertaken by CIs PRW [70] and AL [71]) of promotions of “SC” (advertisements or posts from influencers), producers, and distributors (bars, bottle shops) of NoLos and organizers of alcohol-free spaces, club nights, or events. This will provide knowledge about the techniques that might impact heavy drinkers’ motivations and attitudes to reducing alcohol and the acceptability of the approach to our 4 case study groups of heavy drinkers. We will use a citizen science approach successfully used by our research team previously [72,73], whereby researchers work alongside participants from the 4 case study groups who document content about alcohol reduction (eg, SC digital advertising) they see. Participants will interpret how they perceive the content and record its effect on their perceptions, attitudes, and behaviors toward SC using a method developed by CI PRW [72]. These data will be used in stage 3 to inform options that have relevance, consonance, and specificity for each group.

Recruitment strategies will be tailored for each group, although approaches to recruitment previously proven useful [72] will guide our approach, including targeted Facebook campaigns [74]. All participants will be ≥18 years of age. *Male* c*onstruction workers* will be recruited via Facebook. Given the prior difficulties with recruiting this group experienced by CIs SM and MS and AI AP, we will draw on CI KL’s extensive networks within the construction industry to supplement the campaign. *LGBTQ+ women* will be recruited via Facebook, social networks, and snowball sampling. CIs SM and TH and AI AP have extensive experience recruiting LGBTQ+ women in multiple countries and contexts. Similarly, CI MS is experienced with recruiting hospitality workers via social media, snowball sampling, and hospitality training providers. *Tertiary education students living in regional areas* will be recruited through La Trobe University’s regional campuses, often co-located with TAFEs, led by CI SM and AI GC. All participants will self-report “heavy” or “moderate” drinking. We will recruit 50 members from each case study group across Australia (total n=200) who will create and upload screenshots (5-10; minimum 5) of social media content that they think relates to NoLos or the SC movement to a study portal. Participants will be asked to take additional photographs of NoLos or SC advertising in their local built environment and upload to the study portal—CIs PRW and BL are experienced in social media analysis. This will enable a meaningful and manageable volume of content for analysis [72]. Participants will be provided with examples of content of interest to this study. They will take note of where they saw the image (eg, specific social media platform or location in their local area) and say what they thought of the content by rating it for personal appeal, impact, and intention per the method previously undertaken by CI PRW to understand the impact of alcohol advertising on Australian women [72]. CI MS has also used citizen science to explore digital advertising of alcohol among young adults [73].

### Stage 1b (Months 3 to 12, Led by CIs PRW and MS and Linked to Aim 1)

This stage will involve interviews with (1) producers or suppliers of NoLo products and organizers of alcohol-free spaces, club nights, or events (n=30) and (2) representatives of the SC movement, including social media wellness influencers and online SC community organizations (n=30). Interviews will investigate reasons behind their decisions to promote SC (including through NoLos), if the case study groups identified in this proposal are among their intended audiences, how NoLos and SC are (can be) promoted to case study groups, and the ways they think the approach could be shaped to meet the needs of the case study groups. Recruitment will be through cold calling and snowball sampling. We do not anticipate problems with recruitment, given that, in our pilot study [24], NoLo producers and distributors and SC stakeholders and representatives were willing to participate. Despite assumptions that NoLo producers may feel disinclined to disclose or be “truthful,” the pilot study by CIs PRW and BL [24] found them to be candid about their motivations and marketing campaigns.

### Stage 2 (Months 12-24, Led by CIs PRW, SM, MS, and BL and Linked to Aim 2)

This stage will use qualitative methods to explore heavy drinkers’ openness to SC and viable options (programs, forums, supports) for reducing alcohol consumption among the 4 case study groups: male construction workers (led by CI KL), LGBTQ+ women (led by CI TH), tertiary education students living in regional areas (led by CI SM), and hospitality sector workers (led by CI MS). Research assistants will undertake in-depth interviews with approximately 30 participants from each case study group recruited during Stage 1a (total n=120), noting that sampling will continue until saturation is reached [75]. If we are unable to recruit enough participants, we will replicate the original recruitment strategies used in stage 1a. Previous experience suggests this sample size will allow for noticeable patterns within the data for each case study group. Nondrinkers and light drinkers will be excluded. Some moderate drinkers from each group will be recruited alongside heavy drinkers for variability in order to triangulate observations on the social context of drinking within their particular social world [54]. Interviews will be conducted online and will last approximately 1 hour. Interview questions will probe for knowledge, attitudes, beliefs, and practices around reducing drinking and the SC movement specifically, what (if anything) appeals to them about SC, factors and barriers that impact preparedness to reduce consumption (particularly where this behavior is habitual and or social setting or identity-based), what alternatives to drinking alcohol they perceive to be available to them, and what supports are available or suitable. CIs and AIs will meet in person during stage 2 to check for agreement in coding (each co-coding a selection of transcripts) to improve explanatory rigor. Together, they will collate stage 1 and 2 findings for the citizens’ juries for deliberation in Stage 3.

### Stage 3 (Months 24-36, Led by CIs PRW, AL, and KL and Linked to Aim 3)

Stage 3 will use consensus development methods, including citizens’ juries and deliberative symposia, to co-develop and co-design alcohol reduction options that have relevance, consonance, and specificity for the groups (presenting data to stakeholders from stage 1). Two weeks prior to the juries and symposia, materials will be distributed to orient participants to the research findings (from stages 1 and 2). CI PRW has extensive experience using consensus development methods [62-65].

Citizens’ juries (n=4) will be conducted with participants from each of the 4 case study groups (a jury of 15 participants per case study group) to deliberate on specific policies, activities, or interventions that might “work” to reduce alcohol consumption for them. Previous research conducted by CI PRW using citizens’ juries suggests a group size of 15 will allow for depth and meaningful dialogue [76-78]. Participants (jurors) will be recruited from stage 1b. Citizens’ juries will be delivered via Teams (CI PRW successfully ran online citizens’ juries in his National Health and Medical Research Council [NHMRC] study [79]). Each jury will meet online over a 2-day period to consider the evidence from stages 1 and 2 and to confer and reach consensus about needed and plausible options for leveraging the SC movement toward alcohol reduction within their social worlds. They will then confer about important factors that shape openness to SC and will put forward recommendations for supporting SC toward alcohol reduction. CIs and AIs will participate in the process to bring in knowledge of the literature and real-world practice (per CI MS’s and AI NL’s experiences working in the alcohol treatment sector) on what “works” in harm reduction and clinical prevention to ensure approaches developed are evidence-based. Participants will discuss their knowledge of sobriety movements, alcohol-free spaces (venues), and alcohol substitutes (NoLos) and express their preferences. They will also discuss the possible facilitators and barriers to reducing alcohol consumption or “being SC” (including possible social pressures to drink alcohol rather than substitutes) to best support alcohol reduction in the case study group they represent. This enables agency and decision-making within groups, including potential for advocating for policy change.

Industry symposia (n=4) will be conducted with stakeholders linked to each of the 4 case study groups to discuss feasible strategies for supporting SC and increased access to NoLos within their respective social worlds. We will run specific symposia to deliberate about how to support alcohol reduction for male construction workers (eg, construction companies and professional associations, specifically Chartered Institute of Building, Australian Institute of Building, Housing Industry Association, and Centre for NSW Work Health and Safety), LGBTQ+ women (eg, ACON, LGBTIQ+ Health Australia, Thorne Harbour), hospitality workers (eg, professional associations and unions, such as HospoVoice; hospitality training providers; hospitality well-being organizations like White Jacket Effect, Go Hospo, and Hospo For Life), and tertiary education students in regional areas (eg, Youth Affairs Council, Local Government Associations, local sporting clubs, universities, and TAFEs). Each symposium will meet for 2 hours to 3 hours via Microsoft Teams and will be identified through our extensive professional networks. For example, CI KL has extensive networks, having undertaken Occupational Health and Safety projects within the building and construction industries. CI BL is an executive member of the Women’s Health Research Translation Network with national networks. CI SM and AI GC have extensive youth sector networks and are institutionally connected with La Trobe University, which has regional campuses in Victoria, and CI TH and AI AP have multiple ongoing contacts and affiliations with LGBTQIA+ groups in Australia.

A policy and practice symposium (n=1) will be conducted with SC organizations (eg, Hello Sunday Morning, Sober in the Country) and public health planners or policy makers at local, state, and federal levels (eg, Drug and Alcohol Services SA, VicHealth, Healthway). All CIs and AIs have well-developed networks across government and nongovernmental organizations and will draw on these networks to recruit participants. The half-day symposium will occur online. It will include a presentation of the ideas developed by citizens’ juries and deliberative symposia, followed by discussion of feasible policy options for NoLo marketing, regulation, pricing, and visibility and practice options including new SC programs. CI PRW is highly experienced at moderating “policy fora.” Policymakers are often willing to spend half a day considering research findings and deliberating about possible policy and practice options. This symposium will develop policy options that harness the popularity of the SC movement for alcohol reduction approaches tailored for each case study group.

PhD project 1 (Torrens University funded) will use citizen science methods to examine how the SC movement is “seen” and acted upon by a cross-section of adults (stratified by age, gender, social class, and drinking levels). Similar methods will be used to those used in stage 1a, but the PhD study will include a larger sample and cross-section of participants with differing levels of consumption, who will collect and co-analyze examples from social media (using approaches from netnography [80]), traditional media, and their local environments (eg, advertising in their neighborhoods) that encourage alcohol reduction, SC, and engagement with the broader wellness industry. Data will be analyzed quantitatively (affect reactions, attitudes, and intentions) and qualitatively (meaning, value, and symbolisms). The PhD will be supervised by CIs PRW, MS, and AL.

PhD project 2 (funded by Torrens University Australia) will support a mixed methods study of SC across the general population (ie, not focused on the 4 case study groups), including a national survey of Australians’ attitudes and motivations toward being SC (including their experience with NoLo products, bars, and events and participating in SC alcohol reduction programs). Areas of questioning will cover the SPT domains of meanings (whether there are group-specific ideas or norms about alcohol and sobriety that can be tackled), capabilities (how sociality is enacted in ways that do and do not involve alcohol), and materials (material impediments to taking up SC such as venue options available to group members), which will enable us to identify public health measures in stage 3 (co-design and co-development). In addition, we will conduct an in-depth examination of alcohol-free bars and events, including ethnography and photovoice methods, paying attention to the spatial, affective, and embodied dimensions of SC spaces as well as the experience of being in alcohol events that offer NoLos. This PhD will be supervised by CIs PRW, BL, SM, and TH.

## Results

This research protocol was funded by the NHMRC as part of their Ideas Grants funding scheme (grant ID: GNT2038211). The study has been funded for a 3-year period, starting in July 2025. The study was funded for AUD $802,000 (US $518,384). Human Research Ethics Committee approval has been sought and approved by the Torrens University Human Research Ethics Committee (ID 0467).

## Discussion

### Significance of the Study

The significance of the study is situated in evidence that Australians are open to the idea of needing to reduce alcohol consumption to improve health [38,39]. An openness to SC could make a large and direct impact on reducing population-level alcohol harms by supporting Australia’s heaviest drinkers with reductions. The proposed project will use innovative methods known to be well-suited to complex public health policy issues like alcohol reduction, where stakeholder values may differ [4]. A co-designed approach aligns with broad acceptance that, to increase the efficacy of public health efforts, members of the heavy-drinking groups should be active participants in designing reduction approaches.

### Expected Outcomes From the Study

There is a global gap in the literature about how to reduce alcohol consumption in heavy-drinking social worlds with persistent high consumption rates, particularly ways that are socially appropriate (ie, suitable, practical, feasible). Our project will provide evidence for targeted disinvestment in approaches that are limited in reducing alcohol harms for heavy-drinking groups. It will provide a template for interventions designed to enable reduced drinking within heavy-drinking social worlds with huge potential for scalability of knowledge, expanding the economic, environmental, social, and cultural benefits within and across Australia and internationally. We will undertake a “natural experiment” on the potential to leverage the SC movement—the various online forums and support programs and networks and proliferation of NoLos—to support alcohol reduction in these groups. Key outcomes are world-first: analysis of the “supply side” of SC and NoLos via social media analysis and industry interviews (stage 1), in-depth investigation of potential ways to support reduction in 4 case study groups (stage 2), and a set of co-designed and co-developed alcohol reduction options for each of the 4 case study groups.

### Major Innovations of the Study

To our best knowledge, this is a world-first attempt to investigate the possibilities of SC and co-design and co-develop new strategies to reduce alcohol consumption that have relevance, consonance, and specificity among 4 groups of heavy drinkers. The success of the SC movement shows how social norms and practices that drive heavy drinking can be reshaped to encourage SC, also harnessing cultural developments such as the increasing focus on health and wellness. This includes considerations such as increased availability of NoLos in off and on-premise venues and messaging that focuses on positives of SC (rather than loss or relinquishment) and resonates with the different case study groups by focusing on values that resonate with the group. Our pilot studies with midlife women showed that features of the philosophy of SC, including security, belonging (to a positive movement in place of drinking), and self-determination, are conducive to reducing drinking. SC also includes programs that improve literacy on alcohol harms and increasing social and emotional supports for reductions—it considers the intricate structural elements that influence alcohol and responds to them with something new. Heavy drinkers could be encouraged to consider why drinking is normalized within their social worlds and if this could be different by reaffirming group social connections through nondrinking. For example, new developments around SC and the growth of sober queer cafes and night clubs provides an important opportunity to explore and change heavy drinking cultures in LGBTQ+ social worlds.

Our study is novel in investigating “social worlds” that encourage heavy drinking and shape people’s decisions around alcohol reduction, as previously conceptualized by CIs SM and MS [4,54]. Conventional approaches, which focus on changing individual alcohol behaviors independent of social contexts, have had limited effectiveness for the 4 case study groups [81]. Our conceptual innovation is our focus on alcohol consumption being enmeshed with a series of other social practices and not a discrete standalone behavior—the social nature of alcohol consumption is at the heart of the proposed study. Focusing on the practices of heavy-drinking “social worlds” is effective, albeit underused, and responds to calls to address “Australia’s culture of intoxication” [4]. The proposed project responds by focusing on the social practices that shape the “social worlds” of 4 case study groups where group norms influence heavy drinking and unlock new potential for reducing alcohol consumption.

Alcohol reduction techniques targeted at the individual (eg, persuading behavior change through screening for problematic consumption) require that consuming alcohol is recognized by the individual as being a “problem.” However, the majority (87%) of Australian drinkers consider themselves “responsible drinkers,” even though 68% of Australian drinkers consume ≥11 standard drinks on a “typical occasion” [82]. This leads to alcohol reduction interventions being resisted [83] since the majority of drinkers do not see their behavior as “risky.” Our focus on examining the suitability and adaptability of SC programs and communities and the increased availability of NoLos does not need people to see drinking as “problematic,” since the SC philosophy is about enabling wellness rather than reducing risk of illness—our focus on co-developing alcohol reduction options regard reduced alcohol as an “asset” to living well and enjoyably.

Rather than implementing yet more individualized interventions with limited efficacy for behavior change (a norm for public health measures around alcohol), we will undertake a world-first “natural experiment” alongside the SC movement to develop socially acceptable and feasible alcohol reduction options. Our multidisciplinary study includes innovative methods such as citizen science, social media analyses, citizens’ juries, and deliberative democratic methods, drawing on the expertise of leading international alcohol researchers. These methods have never been used to understand and explore options for alcohol reduction within our 4 case study groups and co-design and co-develop alcohol reduction options.

### Potential Limitations of the Study

This is a very complex study with numerous “moving parts.” We plan to implement a number of highly innovative methods with different population groups of heavy drinkers. A potential limitation is that we do not recruit enough participants in each of the case study groups, although we have excellent professional networks and prior expertise in working with each group over many years. We also have a plan for recruitment from one stage of the research to the next, with a proportion of participants in stage 1 being recruited into stage 2, then a proportion of those being recruited into stage 3. This increases the feasibility of successful completion and outcomes from the study.

## Abbreviations

AI: associate investigator
ARC: Australian Research Council
CI: chief investigator
LGBTQ+: lesbian, gay, bisexual, transgender, queer, plus
LGBTQIA+: lesbian, gay, bisexual, transgender, queer, asexual, intersex, plus
NHMRC: National Health and Medical Research Council
NoLo: no or low-alcohol
SC: sober curious
SPT: social practice theory
TAFE: technical and further education

## Data Availability

The anonymized data sets generated or analyzed during this study may be available from the corresponding author on reasonable request (pending appropriate Human Research Ethics Committee approvals).
